# Manual lateralization in infancy

**DOI:** 10.3389/fpsyg.2014.01575

**Published:** 2015-01-12

**Authors:** Arlette Streri, Maria Dolores de Hevia

**Affiliations:** ^1^University Paris DescartesParis, France; ^2^Laboratoire Psychology de la Perception, Centre National de la Recherche ScientifiqueParis, France

**Keywords:** lateralization, haptic perception, infant, newborn, hemispheric asymmetry, hemispheric specialization

## Introduction

Most people are right-handed and left-cerebrally dominant for language and this brain dissymmetry has been based on “*which*” type of information each hemisphere controls (Corballis, 2012): In general terms, the left hemisphere (LH) is usually specialized in auditory information, specially linguistic stimuli, whereas the right hemisphere (RH) would be specialized in visuo-spatial information. In another approach, brain dissymmetry depends on “*how*” each hemisphere processes information: While the RH would process the visual spatial data in a more global, simultaneous and holistic way, the LH would process the linguistic data in a more analytic, sequential and serial manner.

Here we will focus on the haptic modality because it has a different status from the visual and auditory modalities, which makes of it a special case that can shed light on the origins of manual laterality. In fact, the arm-hand system fulfills both a perceptual function by gathering information on objects, and an instrumental function by transporting objects in space (Hatwell, 1983). Moreover, one of the particularities of the haptic modality during the fine manipulation of objects is that it involves a perceptual-motor coupling difficult to dissociate. Since the arm-hand system is specialized in the treatment of spatial information like the visual modality, the RH should play a dominant role. Because this is a contact modality, in which the hand processes spatial information sequentially like the auditory modality, it would be the LH that should intervene in this function. Notwithstanding the difficulty of disentangling the perceptual and motor aspects from one another, we argue that thanks to the methodological tools used in infancy it is possible to dissociate them. In fact, when holding an object, infants are evaluated in their motor aspects (holding time, strength of the grasp, etc.), while when infants are habituated to an object and then tested on the recognition of its properties, it is the perceptual-cognitive aspects that are revealed with this procedure. Therefore, we assume that the process of lateralization manifests itself in a clear way both from the perception as from the motor function of the hands.

We will review and discuss the literature on the motor aspects of manual asymmetries, and will focus on the methodological importance in order to disclose the perceptual aspects of these asymmetries. Finally, we discuss the manual perceptual asymmetries, and the findings and implications in the context of hemispheric specialization.

## Manual motor asymmetries

From birth onwards, infant's motor asymmetries have been described to predict their laterality as children and adults. Hand and arm movements such as grasping, reaching for objects, unimanual or bimanual holding, head turning and the rooting reflex, postural orientation (ATNR), and stepping reflex have been evaluated for this purpose (Michel, 1988; Provins, 1992; Fagard, 2013). For example, the newborn's grasping reflex in response to a stimulation of the palm of the hand, already present *in utero* (Erhardt, 1973), was considered the best predictor of children and adult laterality (Tan and Tan, 1999).

Studies focusing on infant manual skills that generally record the object holding time, reveal that when we compare holding performance with the right vs. the left hand, there is a superiority of the right hand (Caplan and Kinsbourne, 1976; Petrie and Peters, 1980; Streri and Gouarir, 1996). In accordance to these findings, it has been shown that at 2 months of age the strength of the grasp reflex is significantly greater for the right than the left hand (Petrie and Peters, 1980), and this asymmetry is present at birth (Tan et al., 1992). This asymmetry in favor of the right hand might therefore reflect an early dominance of the LH (Lockman and Wright, 1988), a finding, however, that does not support the idea that the spatial processing is controlled by the RH. Conversely, when the infant holds an object in each hand at the same time, the right hand holds the object for a shorter time than in the one-handed condition, as if it were disturbed by the left hand's simultaneous activity. In fact, the difference in the object holding times, with an advantage for the right hand, only becomes significant from the age of 5 or 6 months (Hawn and Harris, 1983; Streri and Gouarir, 1996). Caplan and Kinsbourne (1976) accounted for these results by suggesting the existence of an inhibition process between the two hemispheres. The LH might be inhibited by the RH when they are activated simultaneously or when they are competing in the same task. However, it has been reported that a left bias exists in the imitation of socially-relevant index finger protrusion. This finding has been interpreted in the context of imitation by reflecting a left-sided mirror neuron system with the potential role of the ipsilateral motor pathway (Nagy et al., 2005). This would suggest that the motor aspects involved in manual activities are related to the LH, and that therefore they are not always established in a strictly contralateral way, but they might depend on the nature of the task.

Tracking the development of manual lateralization is complicated by the fact that neonatal reflexes change either by becoming more mature or by disappearing, and it seems difficult to establish whether they are supported by the RH or LH, or are under subcortical control. Moreover, infant hand-use preferences are too variable and unstable for a valid assessment. The development of right or left biases in handedness of infants and children is fluctuant and rather dynamic, depending on task, posture and motor development. This is the reason why we suggest that the perceptual aspect of the hand could more efficiently reveal the way information is processed by the two hemispheres very early in development.

## How dissociating motor from perceptual aspects?

Is an infant able of processing information about an object's properties when holding or grasping it? Some authors have argued that a newborns' tendency to strongly close the fingers on an object or an adult's finger makes it impossible for them to perceive the fine details of the object, and to recognize an object through a single grasp (Katz, 1925/1989; Roland and Mortensen, 1987). In adults, the tactile information about an object's shape is sampled sequentially by several fingertips sweeping over the object's surface in different directions and at different rates. A newborn's grasping may be insufficient to detect fine features. Still, a series of several grasps might be adequate to perceive the global shape, the rough texture, etc., and to give an involuntary exploratory procedure similar to the adult “enclosure” (Lederman and Klatzky, 1987) that detects the global shape.

Several studies have used the habituation/dishabituation procedure where an object is successively presented to the same hand for several trials (Streri and Pêcheux, 1986; Streri, 1987). After a progressive decrease in the holding time is observed, a new object is placed in the same hand. If an increase in the holding time occurs, this reaction to novelty is taken as evidence for discrimination between the two objects. This procedure, used with newborns, shows that habituation and discrimination are present for both the right and the left hands, and there is no asymmetry between hands (Streri et al., 2000). These findings demonstrate that both hands are able to process information about an object's shape in the same way. Nonetheless, this procedure only reveals a low-level processing of information that is common to all the sensory modalities.

In order to reveal an asymmetry between the two hands, one needs to employ tasks involving higher-level cognitive processes such as cross-modal transfer, memory, and global vs. analytic processing in haptic perception. It is only through these tasks that we can establish that the information is being processed at a cortical level and also that a neonatal reflex is not involved.

## Manual perceptual asymmetries

Some studies have tested cross-modal transfer of shape from touch to vision in newborns with both the right and the left hands. In these studies, after tactile habituation with an object without vision control, newborns were presented with the familiar shape and a novel shape. Newborns showed evidence for visual recognition of the shape after habituation with the right, but not with the left hand, revealing for the first time a perceptual asymmetry between the two hands (Streri and Gentaz, 2004). This unexpected finding, which might be specifically related to the involvement of two modalities, opens the question of which aspects of the task are controlled by each hemisphere. There are several possibilities. According to the information processing hypothesis, the geometric property (or spatial information) would be controlled by the RH. Alternatively, the motor aspect involved in grasping rather than perceptual, would be controlled by the LH, because the strength of the grasp is greater with the right hand. Another possibility is that the sequential aspect, emerging from the multiple grasps taking place during the habituation phase, prevails and it is therefore controlled by the LH (type of processing hypothesis). All these interpretations can be specific at birth and do not predict future handedness. Finally, when using a paradigm where one modality is involved, 2-month-old infants have been shown to retain better the information on object shape with their left (RH) than with their right hand (LH) (Lhote and Streri, 1998), and that in 6-month-old infants the left hand tends to be dominant for perceptual function, while the right hand is preferred for holding objects and trigger directed abilities (Streri and Gouarir, 1996).

However, although these studies show a perceptual asymmetry during the first years of life, they do not shed light on “how” the information is represented in each hemisphere. As for the visual modality, we know that there is a hemispheric specialization in the treatment of visual information, with the LH treating information analytically and the RH globally (Deruelle and de Schonen, 1991). Some studies have addressed this question in the tactile modality since young babies are able to process information on the contour of objects as well as to detect an element inside an object (Bushnell and Boudreau, 1993). In particular, in a discrimination task allowing a global or an analytic processing mode, four-month-old infants explored small objects under two conditions. In the “global condition,” habituation and discrimination were conducted with the contour of the object, with the details remaining identical; in the “analytic condition,” habituation and discrimination were conducted with the details of the object, with the contours remaining identical. This study showed that the haptic mode allows young infants to differentiate in a complex object configural properties from featural details. Even more remarkable is the fact that this differentiation was specific to one hand: While infants detected the change of contour, but not the details, with their left hand (RH), they detected the change of details, but not the contour, with their right hand (LH) (Streri, 2002). Therefore, this haptic specialization process in infancy is parallel to the visual processing in spatial tasks.

## Conclusions

The haptic modality has an intermediate status between the visual and the auditory systems. Moreover, there are both perceptual and motor aspects closely involved in this modality. Although the mechanisms responsible for the relationships between haptic cognition and hemispheric asymmetry in infant development are not yet clearly elucidated, there is some evidence for manual lateralization early in life. However, the studies on the perceptual manual asymmetries in infancy do not predict the handedness in children or adults for writing, painting, or drawing tasks, which are fine motor abilities. Rather, they shed light on which type of processing the two hemispheres are involved when information is gathered by the hands. Moreover, even though it is difficult to dissociate the perceptual aspects from the motor ones during the manipulation of an object, the perceptual studies suggest that when the task is predominantly motoric it is the LH that is in charge, while when the perceptual aspects are dominant it is the RH that prevails. Therefore, these studies reveal a hemisphere specialization during the processing of information in the same task.

### Conflict of interest statement

The Guest Associate Editor Jacqueline Fagard declares that, despite being affiliated to the same institution as the authors, the review process was handled objectively and no conflict of interest exists. The authors declare that the research was conducted in the absence of any commercial or financial relationships that could be construed as a potential conflict of interest.
